# Validation of a culturally modified short form of the McCarthy Scales of Children’s Abilities in 6 to 8 year old Zimbabwean school children: a cross section study

**DOI:** 10.1186/1471-2377-12-147

**Published:** 2012-11-29

**Authors:** Gwendoline Q Kandawasvika, Paul M Mapingure, Margaret Nhembe, Richard Mtereredzi, Babill Stray-Pedersen

**Affiliations:** 1 Department of Paediatrics and Child Health, University of Zimbabwe, Harare, Zimbabwe; 2 Department of Community Medicine, University of Zimbabwe, Harare, Zimbabwe; 3 Department of School Psychological Services, Ministry of Education and Culture, Harare, Zimbabwe; 4 Division of Obstetrics and Gynecology, Rikshospitalet, University of Oslo, Oslo, Norway

## Abstract

**Background:**

The burden of cognitive impairment among school children from developing communities is under reported due to lack of culturally appropriate screening tools. The objective of this study was to validate a culturally modified short form of the McCarthy Scales of Children Abilities (MSCA) in school children aged 6–8 years from varied backgrounds.

**Methods:**

One hundred and one children aged 6–8 years attending mainstream classes were enrolled cross-sectionally from three schools: one rural and two urban. Two assessments were conducted on each child and the Short form MSCA was compared to an independent assessment by the educational psychologist.

**Results:**

When comparing the results of the MSCA to local standard at -2SD, -1.5 SD and -1SD the sensitivity rates ranged from 17 to 50% with lower sensitivity at -2SD cut-off point. Specificity rates had less variation ranging from 95% to 100%. The number of children identified with cognitive impairment using -2SD, -1.5SD and -1SD below the mean for MSCA as a cut-off point were 3(3%), 7(7%) and 13(13%) respectively while the psychologist identified 18 (18%). The overall mean score on MSCA was 103 (SD 15). The rural children tended to score significantly lower marks compared to their peers from urban areas, mean (SD) 98(15) and 107(15) respectively, p=0.006. There was no difference in the mean (SD) scores between boys and girls, 103(17) and 103(15) respectively, p=0.995.

**Conclusion:**

The culturally modified short form MSCA showed high specificity but low sensitivity. Prevalence of cognitive impairment among 6 to 8 year children was 3%. This figure is high when compared to developed communities.

## Background

Children in Africa are at risk of biological and psychosocial insults that affect brain development [1]. The magnitude of neurocognitive impairment among sub-Saharan Africa school children remains underestimated due to lack of culturally sensitive assessment tools. With over 2000 native languages spoken on the African continent, comparison of research findings on child cognitive development conducted across the diverse African cultures remains a challenge due to cross cultural differences. With no data available, advocacy for the primary prevention and early intervention of cognitive impairment receives a lip service from national policy makers.

The choice of a neuropsychological tool depends on the specific disease pathophysiology and anticipated neurocognitive deficits [2,3]. In assessing school children from resource constrained settings, the selected neuropsychological tools should be culturally appropriate, able to identify pathology and easy to administer by primary health care providers. Widely used tools were developed and normed in the developed countries and applicability in African children established in a few research settings [4-8]. In developing communities 2 options exist: development of a new culturally sensitive tool or modification of established Western tool. When a screening tool is adapted, it validity against a set gold standard is assessed by the sensitivity (the percentage of people with the condition who are correctly identified by the instrument as having the condition) and specificity (the percentage of people without the condition who are correctly identified by the instrument as not having the condition). There is no gold standard neuropsychological test. Although it is feasible to conserve construct validity by developing a new tool, [9] the exercise is expensive in resource constrained settings.

The prevalence of cognitive impairment among school children in Zimbabwe is not well defined. A national community survey reported a 3.2% prevalence rate of developmental problems among the under five years children surveyed [10]. As children are at risk for cognitive impairment from a variety of factors including infections, nutritional deficiencies, complications of pregnancy, prematurity and accidental injury; early screening and referral for management and rehabilitation is essential.

The purpose of this study was to validate the culturally modified Kaufman’s short form of the McCarthy Scales of Children’s Abilities (MSCA) in school children aged 6–8 years against the local standard and assess the prevalence of cognitive impairment. We chose to use the short MSCA [11,12], a cognitive test which has been validated in a similar population in South Africa [13], for its easy to administer in a busy ambulatory. The MSCA instrument can be used to track development from preschool age (2.5 years) to school age (8.5 years) [12].

## Methods

Study Design: This was a cross sectional descriptive study conducted at three primary schools in Zimbabwe: one rural area in Goromonzi, one urban low income and 1 urban high income area both in Harare, from July to December 2009.

Study Setting: The population of Zimbabwe is predominantly African (99%) with three official languages namely Shona Ndebele and English [14]. Shona is spoken by the majority (70%) although there are other indigenous languages. Primary school education is offered in one of the three systems, government, church or private. The majority of children (65%) reside in the rural areas and are enrolled in the government system. The primary school participation is over 90% [15] with an adult literacy rate estimated at 90% [14].

Sample size and sampling: A total of 101 Shona speaking school children from the general population aged (72 to 104 months) attending main stream second grade were recruited. Children with severe neurodevelopmental impairment, intellectual, visual or auditory disability were excluded as they usually attend special education classes. The schools were conveniently selected based on geographic accessibility and historical representation of the three types of primary schools currently available in Zimbabwe: urban middle class (Group A), urban low income (Group B) and rural. All registered schools adhere to the same education syllabus.

Measures: We assessed children’s cognitive abilities using the short form MSCA [12] and compared the performance with an educational psychologist assessment, the local standard practice for screening children with suspected cognitive impairment.

### Neuropsychological tests

Short form MCSA: Is an abbreviated six item version of the MSCA’s general cognitive scale. The McCarthy Scales of Children’s Abilities is an assessment tool that was developed for children of ages 2½ through to 8½ years. It assesses children’s present level of functioning in intelligence and motor ability with the aim of identifying possible developmental delay in different skill areas. It consists of puzzle solving, word knowledge, numerical memory, verbal fluency, counting and sorting and conceptual grouping. Based on the sum of the child’s weighted raw score on the six tests, the estimated general cognitive index is computed [12]. The mean for the GCI is set at 100, with a standard deviation (SD) of 16. Although there is controversy on the cut-off point to define cognitive impairment, we opted to use the clinically acceptable cut-off point of -2SD below the mean. The short form MSCA provides proportional representation to the verbal, perceptual performance, qualitative and memory scales and serves as a screening instrument for potential learning disorder. Items from the verbal, perceptual-performance and quantitative are content oriented, with no subset from one domain contributing to the score of another domain. The MSCA was selected for its psychometric properties, appropriateness for children 2½ to 8½ and its history. It is one of the measures used for developmental assessment of preschool and school children in both research and clinical practice.

Education psychologist: In Zimbabwe school age children with suspected cognitive impairment are referred to the School Psychological Services for screening. As the local standard of practice, the Educational psychologists assess the children using adapted psychometric tools. Depending on the reason for referral children over 7 years of age are screened for reading readiness, oral spelling test, basic number skills and mental age determination. The psychologist assessed basic arithmetic skills with the British abilities scale (BAS) [16] and word building skills with Daniels and Diack’s graded spelling test. The BAS utilizes a basal and ceiling format for testing (Basal: 3 or fewer passes within an 8-item set; Ceiling: 5 or fewer passes within the 8-item set. The Daniels and Diack assesses reading, word recognition and building competence [17].

Each child was presented with a word identifying subset made up of word reading list of increasing difficulty and was required to read as many words from the list. A graded spelling test to evaluate word building skills according to the Daniels and Diack graded spelling test was administered [17] to the whole class. Academic competence was computed from a table of norms. The cut-off point for developmental delay was academic performance that fell below 2 chronological years for age. In the local practice, the educational psychologist’s assessment identifies if a child has normal function, requires remedial in either reading or mathematics or recommends transferred from the mainstream class to a remedial class for children with global cognitive delay. The students with special needs are grouped for special class services according to similarity of educational needs and students’ classroom management plan. For this study the educational psychologists’s assessment served as our gold standard. Two categories of children were therefore identified; those with normal development or children with global developmental delay.

### Validation process

#### Modifications

Test items were examined individually to establish which pictures or items were recognizable and to evaluate the clarity of instructions. After piloting, a focused group discussion between teachers and the research team reached a consensus on the items to be substituted with familiar materials. Culturally appropriate modifications to some of the test items were made to render the battery of tests applicable after pretesting. The picture of a sailboat was not familiar with the rural children and was replaced with a similar coloured picture of a lorry. The term pennies were replaced with cents, cookies with biscuits and syrup with porridge. Test content, format and test order was preserved. Children could recognize the substituted items easily and the overall testing time was shortened.

Data was reviewed regularly to optimise quality control. Inter-observer and inter-tester reliability was maintained by centralized training of all the project examiners by a senior clinical psychologist. Translation of the MSCA into the local language was done by the research team with the help of linguists. The translated Shona version of the MSCA was translated into English by a professional translator and members of the research team fluent in both languages. The back translated English version and the original instruments were reconciled. Inter- rater reliability was enhanced by strict adherence to standardised the scoring system according to the MSCA manual. The examiners were trained in the test administration and test set order.

### Criterion validity

The sensitivity and specificity of the screening instrument was tested by investigating the association between the number with scores below 2SD, 1.5SD, 1SD of the GCI and global developmental impairment according to the educational psychologist assessment.

Each child’s cognitive abilities was assessed independently by two examiners: the researcher and the educational psychologist within a 24 hour period. The children were counter balanced between the two examiners with the first half examined by the researcher first while the other was examined by the educationist first. A swop over was made the next day where the first half was examined by the educationist and visa versus. The researcher was trained by and supervised by a licensed clinical psychologist. After establishing rapport the, MSCA assessments took 12 to 15 minutes depending on the child’s competency.

### Data analysis

We calculated the sensitivity and specificity, the positive and negative predictive values, and over and under referral rates for the MSCA based on cross tabulations of the educational psychologist’s assessment and the MSCA screening. Raw agreement between the 2 observers (chance corrected agreement) was calculated using the kappa coefficient [18]. Cognitive function was classified according to the sum of the weighted scores in the six tests items of the Kaufman short form of MSCA. We selected 3 cut- off points for cognitive impairment: -2SD, -1.5SD and -1SD in this cohort. All analysis was conducted using SPSS for windows (Rel 12.0.1, 11 Nov 2003, Chicago, SPSS, Inc).

Ethical clearance to interview the children was sought from the provincial school psychological department, provincial education department. The general overview of the study and entire voluntary nature of participation was disclosed to the primary care givers before obtaining an informed verbal consent. Verbal assent was obtained from each child. The study was approved by the medical research council of Zimbabwe.

## Results

We conducted direct assessment of 101 children whose median (range) age was 97 (77–102) months and of whom 60 were female. Distribution of participants by site was 40(40%) rural, 37(37%) urban low income and 24(24%) urban high income. All the children had attended preschool.

Table 1, 2 and 3 show the screening results obtained when comparing the results of the MSCA to local standard at -2SD, -1.5 SD and -1SD. The sensitivity rates were low, ranging from 17 to 50% with lower sensitivity at cut-off -2SD. Specificity rates had less variation ranging from 95% to 100%. The under referral rate was 15% at -2SD which meant out of the 101 children assessed 15 would fail to be identified by the MSCA when they have cognitive impairment. The positive predictive values ranged from 69 to 100% whilst the negative predictive values had less variation and ranged from 84 to 89%.

**Table 1:** Contingency table for the observer agreement for cognitive impairment at cut-off −2 SD

**Assessment results cut-off −2 SD**			
**Observer 1**			
Observer 2	Delayed	Not Delayed	Total
	A	B	
	True positive	False positive	
	3	0	3
	C	D	
	False negative	True negative	
	15	83	98
Total	18	83	101

Sensitivity = A / A + C 3/18=17%.

Specificity = D / B + D 83/83=100%.

Total Agreement = A + D / A + B + C + D (3+83)/101=86%.

Positive Predictive Value = A / A + B 3/3=100%.

Negative Predictive Value = D / C + D 83/98=84%.

Over-referral = B / A + B + C + D 0/101=0%.

Under-referral = C / A + B + C + D 15/101=15%.

Likelihood ratio—positive = Sensitivity / (1 – specificity) incalculable.

Likelihood ratio—negative = (1 – sensitivity) / specificity .83.

**Table 2:** Contingency table for the observer agreement for cognitive impairment at cut-off −1.5 SD

**Assessment results cut off −1.5 SD**			
**Observer 1**			
Observer 2	Delayed	Not Delayed	Total
	A	B	7
	True positive	False positive	
	7	0	
	C	D	94
	False negative	True negative	
	11	83	
Total	18	83	101

Sensitivity = A / A + C 7/18=39%.

Specificity = D / B + D 83/83=100%.

Total Agreement = A + D / A + B + C + D (7+83)/101=89%.

Positive Predictive Value = A / A + B 7/7=100%.

Negative Predictive Value = D / C + D 83/94=88%.

Over-referral = B / A + B + C + D 0/101=0%.

Under-referral = C / A + B + C + D 11/101=11%.

Likelihood ratio—positive = Sensitivity / (1 – specificity) incalculable.

Likelihood ratio—negative = (1 – sensitivity) / specificity .61.

**Table 3:** Contingency table for the observer agreement for cognitive impairment at cut- off -1SD

**Assessment at cut off – 1SD**			
**Observer 1**			
Observer 2	Delayed	Not Delayed	Total
	A	B	13
	True positive	False positive	
	9	4	
	C	D	88
	False negative	True negative	
	9	79	
Total	18	83	101

Sensitivity = A / A + C 9/18= 50%.

Specificity = D / B + D 79/83=95%.

Total Agreement = A + D / A + B + C + D (9+79)/101=87%.

Positive Predictive Value = A / A + B 9/13=69%.

Negative Predictive Value = D / C + D 79/88=89%.

Over-referral = B / A + B + C + D 4/101=4%.

Under-referral = C / A + B + C + D 9/101=9%.

Likelihood ratio—positive = Sensitivity / (1 – specificity) 10.

Likelihood ratio—negative = (1 – sensitivity) / specificity 0.53.

Total agreement and kappa values between the two assessments on the presence of cognitive impairment at cut off point -2SD,-1.5 SD and −1 SD below the mean respectively was 0.86 and 0.247 indicating fair agreement; 0.90 and 0.511 indicating moderate agreement; 0.87 and 0.507 indicating moderate agreement.

The number of children identified with cognitive impairment using -2SD, -1.5SD and -1SD below the mean for MSCA as a cut-off point were 3(3%), 7(7%) and 13(13%) respectively while the psychologist identified 18 (18%). The overall mean (SD) score on MSCA was 103 (15). The rural children tended to score significantly lower marks compared to their peers from urban areas, mean (SD) 98(15) and 107(15) respectively, p=0.006. There was no difference in the mean (SD) scores between boys and girls, 103(17) and 103(15) respectively, p=0.995.

## Discussion

The present study was undertaken among school children from Zimbabwe to validate the culturally modified short form of the Kaufmann MSCA as a screening tool for cognitive impairment. Cognitive development is not routinely assessed in clinical practice due to unavailability of culturally appropriate tools and trained psychometric assessors. Previous validation studies differ on the definition of developmental impairment making direct comparison difficult [19]. When the set cut off point is -1SD, children with both mild to severe impairment are captured, leading to early referral for those with mild impairment [20].

Researchers concur that a satisfactory screening tool should have both sensitivity and specificity of at least 70% [21,22]. Previous research emphasizes the importance of the selected cut-off point in assessing the validity of a screening instrument [22,23]. We compared how the short MSCA was performing at -2SD, -1.5 SD and -1SD below the mean. The sensitivity rates for the MSCA were low (50%, 38% and 17%) compared to the specificity rates which were high (95%, 100% and 100%) when using the more stringent cut-off for the standardized assessment of -2SD. It is plausible that the short form MSCA does not contain enough items to capture the diverse cognitive problems with equivalent precision. A low sensitivity in a developmental screening tool may provide a false assertion to parents and guardians who would otherwise benefit from referral. With such low sensitivity, the universal use of the translated MSCA among Shona speaking children should be applied with caution.

In the context of this study, we found that agreement between the two definitions of cognitive impairment was moderate (using kappa) at -2SD. In clinical practice, it is prudent to link the screening tool to the disease pathophysiology. In the contests of a myriad of biological and environmental factors that potentially negate cognitive development in school children such as infective, environmental, nutritional causes, a simple screening tool will estimate the burden of cognitive impairment and inform policy makers. A study from Pakistan reported prevalence of mild mental retardation among 6–10 year olds of 6.1% [24] whilst a study from Yemen reported prevalence of 15.7%. A report from a population based survey in Metropolitan Atlanta estimated the prevalence of mental retardation at 10.3 per 1000 in 10-year-old children [25]. It is plausible that the disruption of schools’ educational curriculum that occurred as a result of the economic challenges the country was going through at the time of the study might have indirectly contributed to the high prevalence of cognitive impairment among study participants.

The identification of children with severe cognitive impairment facilitates referral to schools that have a special education curriculum. In developed communities, referred children benefit from individualized cognitive stimulation programmes especially designed to promote student success and achievement. In Zimbabwe although a special education curriculum exists for visual , hearing or cognitive impairment; the classes are congested, are available in private expensive institutions and characterized by a high special education trained teacher shortage [26]. Community based strategies employed elsewhere include integration of children with mild cognitive impairment into normal schools. Detection of intellectual disability is stigmatizing in developing communities [27]. It is essential to guard against stigma by increasing the public awareness of the condition , making screening routinely available to all children at scheduled periods and explaining the benefits of such programs such as the promotion of literacy. Parental compliance with screening protocols is enhanced if stigma is minimised.

In the context of a developing community, screening with MSCA would identify 3/18 7/18 and 9/18 using 2SD 1.5 SD and 1 SD respectively of children with mild to severe impairment who might not have been identified and would serve as an entry point towards a comprehensive primary prevention strategy that seeks to promote early childhood development.

We acknowledge our sample was small compared to the standardization population; however the study generated baseline data on the use of an adapted developmental tool in Shona speaking school children and may supplement existing information on the cognitive development.

When the MSCA validated in South Africa by Ritcher et al., they reported on the predictability validity of MSCA with school achievement and good discrimination between learning disabled children and normal children. We found high positive predictive values (probability that a child who screens positive actually has a cognitive impairment) ranging from 69 to 100%. The negative predictive values reported in this paper support the ability of the tool to discriminate normal children from those with impairment. Since both positive and negative predictive values are impacted by the prevalence of cognitive impairment in the population under study, this may suggests high prevalence of developmental impairment in the sample studied. Population screening may prove to be cost effective for this community.

For most clinicians in resource restricted settings the ability of a tool to over or under refer has a direct bearing on the already stretched resources. The under referral rate was high (proportion of all children who screen negative when they actually have a developmental delay) and ranged from 9 to 15% in this study.

We had the following limitations in this study. A single criterion measure was used. We did not evaluate motor, emotional development, or activities of daily living. We did not administer a supplemental parental or guardian completed screening might have painted a more complete picture. The unavailability of a culturally appropriate developmental instrument to compare as a standard was a limiting factor in this study. Adaptation we made to the short form of MSCA might have influenced the reliability of the test score as some cultural bias in the MSCA might have remained. We did not study the inter-rater reliability using Intra-class Correlation.

From our experience in this study, we learnt that it is possible to adapt components of an established psychometric tool such as MSCA to suit local culture and practice. We were able to conduct a study among children from a general population. The strength of our findings was in the estimation of the prevalence rates of learning disabilities among rural and urban 6–8 year old children after the translation of a commonly used psychometric tool into Shona language.

## Conclusion

The culturally modified short form MSCA showed high specificity but low sensitivity. Prevalence of cognitive impairment among 6 to 8 year children was 3% at 2SD below the mean according to MSCA. This figure is high when compared to developed communities justifies a larger study among children of various age groups.

## Competing interests

The authors declare that they have no competing interest.

## Authors’ contributions

GQK involved in the conception, design, assessment on the participants, drafting of manuscript. MN involved in the standardization of tool and assessment of participants. MPM involved in the statistically analysis and interpretation of the results. BSP involved in the interpretation of data and revision of manuscript. All authors read and approved the final manuscript.

## Pre-publication history

The pre-publication history for this paper can be accessed here:

http://www.biomedcentral.com/1471-2377/12/147/prepub
